# Triple-network analysis of Alzheimer’s disease based on the energy landscape

**DOI:** 10.3389/fnins.2023.1171549

**Published:** 2023-05-23

**Authors:** Youjun Li, Simeng An, Tianlin Zhou, Chunwang Su, Siping Zhang, Chenxi Li, Junjie Jiang, Yunfeng Mu, Nan Yao, Zi-Gang Huang, Alzheimer’s Disease Neuroimaging Initiative

**Affiliations:** ^1^The Key Laboratory of Biomedical Information Engineering of Ministry of Education, Institute of Health and Rehabilitation Science, School of Life Science and Technology, Xi'an Jiaotong University, The Key Laboratory of Neuro-informatics and Rehabilitation Engineering of Ministry of Civil Affairs, Xi'an, Shaanxi, China; ^2^Research Center for Brain-inspired Intelligence, Xi'an Jiaotong University, Xi'an, Shaanxi, China; ^3^Department of Military Medical Psychology, Air Force Medical University, Xi'an, Shaanxi, China; ^4^Department of Gynecological Oncology, Shaanxi Provincial Cancer Hospital, Xi'an, China; ^5^Department of Applied Physics, Xi'an University of Technology, Xi'an, China; ^6^The State Key Laboratory of Congnitive Neuroscience and Learning, Beijing Normal University, Beijing, China

**Keywords:** triple-network, energy landscape, Alzheimer’s disease, resting-state fMRI, dynamics analysis

## Abstract

**Introduction:**

Research on the brain activity during resting state has found that brain activation is centered around three networks, including the default mode network (DMN), the salient network (SN), and the central executive network (CEN), and switches between multiple modes. As a common disease in the elderly, Alzheimer’s disease (AD) affects the state transitions of functional networks in the resting state.

**Methods:**

Energy landscape, as a new method, can intuitively and quickly grasp the statistical distribution of system states and information related to state transition mechanisms. Therefore, this study mainly uses the energy landscape method to study the changes of the triple-network brain dynamics in AD patients in the resting state.

**Results:**

AD brain activity patterns are in an abnormal state, and the dynamics of patients with AD tend to be unstable, with an unusually high flexibility in switching between states. Also , the subjects’ dynamic features are correlated with clinical index.

**Discussion:**

The atypical balance of large-scale brain systems in patients with AD is associated with abnormally active brain dynamics. Our study are helpful for further understanding the intrinsic dynamic characteristics and pathological mechanism of the resting-state brain in AD patients.

## 1. Introduction

Alzheimer’s disease (AD) is a common degenerative neurological disease that typically begins with memory loss and progresses to impairments in a series of higher cognitive functions, followed by a loss of ability to live independently and eventual death. The onset of AD is obscure and seriously affects the normal life of patients. The etiology and specific pathogenesis of the disease have not been fully elucidated (Delbeuck et al., 2003; Cummings et al., 2019; Lei et al., 2021). Therefore, methods designed to better understand the pathogenesis of AD and identify specific brain abnormalities in the early stages of AD are crucial.

Resting-state fMRI reflects the spontaneous activity of neurons when the brain is not performing a task. It has been widely employed in studies of a variety of neurological and psychiatric disorders. Because it does not require subjects to perform any task, resting-state fMRI is ideal for AD patients with cognitive decline (Vemuri et al., 2012; Yang et al., 2020). Different functional connectivity networks have been described based on the synchronization of low-frequency BOLD signals in the resting state (Damoiseaux et al., 2006). Vinod Menon proposed that among the inherent functional networks in the human brain, the default mode network (DMN), the salient network (SN), and the central executive network (CEN) are particularly crucial (Menon, 2011). The DMN is more active in the resting state, while the DMN is inhibited instead during the execution of tasks. In contrast, the CEN is less activated in the resting state and more activated when subjects are performing tasks or receiving external stimuli. The SN is generally considered to coordinate the DMN and CEN (Buckner et al., 2008; Lerman et al., 2014; Liao et al., 2021). Therefore, some studies have proposed the three networks as a “triple-network” model, which suggests that these three networks play an important role in the functions related to cognitive tasks performed by the brain, and they are considered the core networks related to the cognitive functions of the brain (Menon, 2011). The “triple-network” model is widely used to study various diseases. Previous studies have found that these three networks are closely associated with cognitive impairment in AD patients, and all three networks are damaged to varying degrees by the disease (Balthazar et al., 2014).

In a conventional resting-state fMRI analysis, functional connectivity is the most commonly investigated metric, which assumes that the BOLD signal is temporally stationary within the scan duration (Greicius et al., 2003). Actually, the brain is a complex and dynamic system with important features such as self-adaptation, self-organization, and multi-stability. A previous study indicated that the activity patterns of the resting brain are presumably in a nonequilibrium process of continuous switching between multiple states and show considerable variability on different time scales (Yao et al., 2020). Revealing the dynamic mechanism of spontaneous neural activity in the resting-state brain has important scientific importance for understanding the working mechanism of the brain and has prospective clinical applications in the prevention and treatment of brain diseases, including AD.

Currently, dynamic functional connectivity (dFC) based on sliding window correlation (SWC) and co-activation patterns (CAP) are two popular methods for AD dynamics analysis. The former was based on dynamically intercepting signals through windows of specific length and then performing functional connectivity analysis within each dynamic window. In recent years, a number of studies have been devoted to the extraction of high-level features from the dFC to achieve effective filtering of invalid information in the dFC and extraction of dynamic change features associated with the disease, contributing to exploring abnormal brain function networks and improving the classification accuracy of AD (Sendi et al., 2021; Gao et al., 2022; Matsui and Yamashita, 2022; Qiao et al., 2022; Ghanbari et al., 2023; Penalba-Sánchez et al., 2023). The principle of CAP was to extract co-activation patterns in certain specific peak points of the BOLD signal time series using a clustering algorithm. By analyzing the spatio-temporal features of these patterns to reveal the underlying mechanisms of abnormal brain function in AD patients, providing some potential biomarkers for the diagnosis and treatment of AD (Ma et al., 2020; Adhikari et al., 2021). The methods and conclusions of recent studies on the dynamic brain function network analysis of AD are shown in Table 1.

**Table 1:** Dynamic brain function network analysis method for AD.

Target	Authors	Method	Conclusions
AD/NC	Gao et al. (2022)	dFC	Extraction of potential advanced features enhanced the classification and diagnosis of brain diseases.
AD/MCI/NC	Ghanbari et al. (2023)	dFC	Redundancy could provide a basis for neuroprotective mechanisms of cognitive ageing and act as an early biomarker of AD.
vmAD/NC	Sendi et al. (2021)	dFNC	From NC to AD, the connectivity strength changed and the temporal properties of the FNC also became dysregulated.
AD/ASD	Qiao et al. (2022)	dFC	Inter-component dFC could be used as a biomarker for the diagnosis of AD et al. and provide a basis for brain connectomics.
AD/MCI/NC	Ma et al. (2020)	CAP	The DMN and visual network of AD are impaired, and transition and CAP entropies can be used as new biomarkers.
AD/NC	Adhikari et al. (2021)	CAPs	Resting-state co-activation patterns could be a biomarker for the diagnosis and prediction of AD.

The regularities presented by activity patterns in the resting brain and the underlying mechanisms are also well suited for research using statistical-physical and nonequilibrium dynamics approaches (Huang et al., 2020). One of the commonly used methods is energy landscape analysis, a data-driven approach based on statistical mechanics (Watanabe et al., 2014; Ezaki et al., 2017; Gu et al., 2018; Kang et al., 2019). This method is similar to CAP that focuses on the nonequilibrium process of switching between resting-state active modes in the brain, but the energy landscape analysis better describes this process by constructing an energy topography of the state space in the brain system based on the statistical distribution, whose structure represents the stability and interrelationship of the system states and reflects the dynamic characteristics of the system in more detail.

In the present study, we extracted a time series based on representative ROIs in brain regions of the triple networks and used the energy landscape approach to analyze the dynamic characteristics of AD patients in the resting state. Some common dynamic indicators were used to characterize the resting triple-network dynamic system in the subjects and correlate it with the clinical index to further explore the dynamic characteristics of the three resting networks and the hidden neural mechanisms of AD in patients, providing some theoretical inspiration for the prevention, diagnosis and treatment of diseases. Finally, the random walk method was used to simulate the dynamic changes in activity patterns in the triple network as a method to verify the effectiveness of the energy landscape analysis method.

## 2. Materials and methods

### 2.1. Subjects

The fMRI data from all subjects investigated in this study were obtained from the Alzheimer’s Disease Neuroimaging Initiative (ADNI) database and were acquired using a Philips 3.0 T MRI instrument.^1^ The downloaded data from each subject included 3 T structural fMRI data and behavioral data. This study included two groups of subjects selected based on the following criteria: (1) 33 patients with confirmed AD and MMSE scores = 6–26 points who were able to complete the entire data collection process and maintain a stable resting state during the entire time and (2) 39 normal healthy elderly people with MMSE scores = 26–30 points, without depression or other types of dementia, and no history of receiving medication for psychiatric diseases.

Resting-state fMRI data were obtained from scans acquired with a 3.0 T Philips instrument. The acquisition parameters were as follows: (1) parameters of the fMRI scan were EPI fast imaging sequence, flip angle = 80°, matrix = 64 × 64 × 6,720, slice thickness = 3.3 mm, TR = 3,000 ms, TE = 30 ms, and pixel spacing = 3.3 × 3.3 × 3.3 mm^3^. (2) Scan parameters of the 3D-weighted T1 structural images were an acquisition plan = sagittal, flip angle=9°, and matrix = 256 × 256 × 170.

### 2.2. Data preprocessing

We preprocessed the resting-state fMRI data using the FSL toolkit and the AFNI toolkit. The preprocessing steps are described below. (1) The first four time points were deleted to ensure that all data were derived from a stable magnetic field. (2) Head movements were corrected. (3) Gaussian spatial smoothing was performed with a half-peak width of 6 mm. (4) Bandpass filtering was performed at 0.01–0.1 Hz. (5) Linear registration FLIRTt with the MNI152 standard spatial template was conducted. (6) White matter and cerebrospinal fluid signals were removed.

### 2.3. Triple-network ROI time series extraction

The ROI template used in this study was obtained from the Neurofunctional Imaging of Mental Disorders Laboratory at Stanford University.^2^ We used this ROI template to extract time series of representative brain regions within the three networks. We selected representative ROIs from the three networks to extract their time series. The coordinates of the selected ROI corresponding to the Brodmann area (BA) and the MNI standard space are shown in Tables 2–5.

**Table 2:** Demographic and clinical scale information of subjects.

	AD (*n* = 33)	NC (*n* = 39)	*p*-value
Gender(female/male)	14/19	16/23	0.90
Age(mean ± SE)	73.35 ± 1.30	74.44 ± 1.07	0.51
MMSE(mean ± SE)	22.97 ± 0.42	28.87 ± 0.20	< 0.001
CDR(mean ± SE)	0.76 ± 0.08	0.00 ± 0.00	< 0.001

**Table 3:** Brain coordinates in the DMN.

Number	ROI	Brain area	BA	MNI space		
				x	y	z
1	dDMN_1_ROI	Left Medial Frontal Gyrus	10	0	49	12
2	dDMN_2_ROI	Left Angular Gyrus	39	−48	−73	32
3	dDMN_3_ROI	Right Media Frontal Gyrus	8	18	38	51
4	dDMN_4_ROI	Precuneus	31	0	−57	30
5	dDMN_5_ROI	Cingulate Gyrus	24	0	−17	35
6	dDMN_6_ROI	Right Angular Gyrus	39	48	−66	29
7	dDMN_7_ROI	Thalamus	/	−6	−6	3
8	dDMN_8_ROI	Left Parahippocampal Gyrus	35	−24	−37	−9
9	dDMN_9_ROI	Right Parahippocampal Gyrus	35	24	−21	−23

**Table 4:** Brain coordinates in the CEN.

Number	ROI	Brain area	BA	MNI space		
				*x*	*y*	*z*
1	LCEN_1	Left Precentral Gyrus	9	−35	21	3
2	LCEN_2	Left Middle Frontal Gyrus	10	−44	46	1
3	LCEN_3	Left Inferior Parietal Lobule	40	−44	−65	4
4	LCEN_4	Left Middle Temporal Gyrus	21	−65	−38	12
5	RCEN_1	Right Precentral Gyrus	9	32	26	4
6	RCEN_2	Right Middle Frontal Gyrus	10	35	62	7
7	RCEN_3	Right Inferior Parietal Lobule	40	46	−54	49
8	RCEN_4	Right Middle Frontal Gyrus	8	3	36	44

### 2.4. Energy landscape analysis method

The energy landscape approach aims to study the interactions among local brain regions from a statistical-physical perspective. The definition of energy can be used to describe the state of a brain system composed of different brain regions. This state model is essentially determined by the empirical distribution of fMRI data in different brain regions. Based on the model defined by the energy function, the energy landscape of the brain system in the state space can be obtained. The structure of the energy landscape reflects the stability and interactions of the states of the brain system. Furthermore, the energy landscape can also reflect a series of dynamic characteristics of the system.

We fit the subjects’ fMRI data to the pairwise maximum entropy model (MEM) using the criteria described below (Ezaki et al., 2018). Due to the large amount of data required for this method, we collected the fMRI signals from the same group of subjects and then performed pairwise MEM fitting. As each network contains a certain number of ROIs, we labeled each ROI as *i* (i=1,2.....) and denoted the binarized activity of the fMRI sequence at moment t as$\;\sigma_{i}^{t}\left(1\leq t\leq T\right)$, with +1 and − 1 representing the activation and deactivation states, respectively. We set a threshold value for each ROI of each subject, and we considered the ROI in an active state when it exceeded this threshold value. The threshold is the average signal value of the ROI for the subject over the whole time course, and thus the ROI of each subject is active approximately 50% of the time moments. The pattern of activity at moment t is represented by the $N_{ROI}$-dimensional binary vector$\;\left[\sigma_{1}^{t},\sigma_{2}^{t},....,\sigma_{N_{ROI}}^{t}\right]$, and there exist $2^{N_{ROI}}$possible patterns of ROI activity, which are enumerated as$V_{1}=\left[-1,-1,...,-1\right]......V_{2^{N_{ROI}}}=\left[1,1,...,1\right].$

For each ROI, we aggregated the data from the same set of subjects according to the time scale and calculated the occurrence frequency of each activity pattern $V_{k}\left(k=1,...,2^{N_{ROI}}\right)$, denoted as $P\left(V_{k}\right)$. In the paired maximum entropy model, the frequencies conform to the Boltzmann distribution as follows:


(1) $$P\left(V_{k}\right)=e^{-E\left(V_{k}\right)}/\sum_{i=1}^{2^{N_{ROI}}}e^{-E\left(V_{i}\right)}$$

where $E\left(V_{k}\right)$ represents the energy of the active mode $V_{k}$, which we calculate using the following equation:


(2) $$E\left(V_{k}\right)=-\sum_{i=1}^{N}h_{i}\sigma_{i}-\frac{1}{2}\overset{N}{\underset{i=1}{\sum }}\;\sum_{\begin{array}{l} i=1\\ j\neq i \end{array}}^{N}J_{ij}\sigma_{i}\sigma_{j}$$

The fitting parameters $h_{i}$ and $J_{ij}$ denote the tendency of the ith ROI to be active when it is isolated and the strength of the interaction between the ith ROI and the jth ROI, respectively. Based on this definition, a smaller energy value corresponds to a greater frequency of occurrence of a pattern of activity on the time scale.

We obtain $h_{i}$ and $J_{ij}$ $\left(i,j=1,2,....,N_{ROI}\right)$by initially calculating the mean and two-by-two correlations for each state in the fMRI data from the subjects. The formula used for the calculation is as follows (where 〈 〉 denotes the average value):


(3) $$\langle \sigma_{i}\rangle =\left(\frac{1}{T}\right)\sum_{t=1}^{T}\sigma_{i}^{t}$$

(4) $$\langle \sigma_{i}\sigma_{j}\rangle =\left(\frac{1}{T}\right)\sum_{t=1}^{T}\sigma_{i}^{t}\sigma_{j}^{t}$$

For a particular $h_{i}$ and $J_{ij}\left(i,j=1,2,...,N_{ROI}\right)$, the mean and correlation expected from the model in equation (1) are as follows:


$$\begin{array}{l} {\langle \sigma_{i}\rangle }_{m}=\sum_{k=1}^{2^{N_{ROI}}}\sigma_{i}\left(V_{k}\right)P\left(V_{k}\right)\;\\ {\langle \sigma_{i}\sigma_{j}\rangle }_{m}=\sum_{k=1}^{2^{N_{ROI}}}\sigma_{i}\left(V_{k}\right)\sigma_{j}\left(V_{k}\right)\;P\left(V_{k}\right) \end{array}$$

Notably, only the information on the average activity in each brain region and the coactivity between two brain regions in the fMRI data were used in the calculation. Other information was not considered in the model, such as information on the coactivity patterns between multiple brain regions. We must calculate the relative entropy of the model distribution of the brain system states and their empirical distribution, which is the K-L divergence (Kullback–Leibler divergence). The model distribution is defined as the Boltzmann distribution, and the empirical distribution is the actual probability distribution of each state in the fMRI data. The difference between the two can be measured using the following K-L divergence $D_{2}$:


(5) $$D_{2}=\sum_{k=1}^{2^{N_{ROI}}}P\left(V_{k}\right).\log_{2}\left(P\left(V_{k}\right)/P_{model}\left(V_{k}\right)\right)$$

We iteratively adjust the values of $h_{i}$ and $J_{ij}$ according to $h_{i}^{new}=h_{i}^{old}+\alpha \left(\langle \sigma_{i}\rangle -{\langle \sigma_{i}\rangle }_{m}\right)$ and $J_{ij}^{new}=J_{ij}^{old}+\alpha \left(\langle \sigma_{i}\sigma_{j}\rangle -{\langle \sigma_{i}\sigma_{j}\rangle }_{m}\right)$, where α is the iteration step, until they gradually approach the values ${\langle \sigma_{i}\rangle }_{m}$ and ${\langle \sigma_{i}\sigma_{j}\rangle }_{m}$ given in eqs. (3, 4) of the model.

The principle of this iteration is based on the gradient descent method, which minimizes the given K-L scatter, improves the accuracy of the model fit by iteratively training the two parameters h and j continuously, and finally obtains an optimized model. The energy landscape of the system is constructed based on this optimized model. The dynamics of different states of the brain system can be characterized by the local minimum, basin, dis-connectivity graph and energy barrier in the energy landscape.

In the energy landscape, among the $2^{n}$ state vectors constructed, the two states in which only one element differs are considered adjacent states, such that each state has *n* neighboring states. When the energy value of a state is less than all *n* neighboring states, the state is defined as a local minimal state. It is also possible to construct disconnected diagrams to represent the main features of each state, including information such as the energy of local minimal states and the energy barrier of switching between neighboring states. The disconnected graph is a tree-like branching graph structure, where different branches represent different local minima, and the branch heights between neighboring local minima represent the energy barriers between them. The disconnected diagram is a more concise and intuitive representation of the relationship between the substable states of the system compared to the energy landscape. The detailed steps used to construct it are as follows:

1. First, the energy landscape is constructed based on the fitted optimization model and visualized to some extent in the superlattice diagram. Each state has a corresponding energy location and is connected to neighboring states, constituting multiple energy basins.
2. Second, the energy maximum corresponding to all current states is set as the energy threshold, which is denoted as Eth.
3. Then, the states and corresponding edges in the superlattice diagram with energies larger than the threshold Eth are removed.
4. Finally, all local minimal states in the superlattice diagram are checked to confirm that they have at least one path to achieve an interconnection.
5. Steps (3) and (4) are repeated and the threshold Eth is set to the maximum energy value in the remaining states, repeating this process until all local minimal states are disconnected from each other.
6. The energy threshold Eth corresponding to the first disconnection of each two local minimal states is recorded, and this value is the energy barrier between these two states, which is the height of the potential barrier between them in the disconnection diagram. As a result, the disconnected graph of local minimal states is obtained.

**Figure 1:**
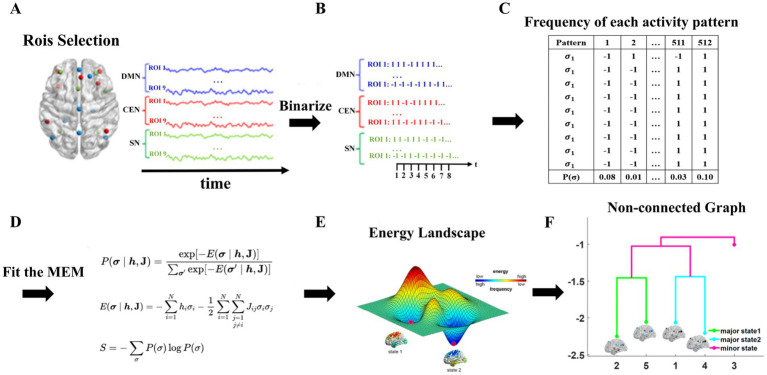
The process of energy landscape analysis. **(A)** extraction of ROI time series, **(B)** binarization of data, **(C)** calculation the frequency of appearance in each activity pattern, **(D)** fitting MEM **(E)** construction of energy landscape **(F)** construction of non-connected graph.

The energy landscape analysis method is a calculation method that can directly interpret multivariate time series. The analysis consists of the following four main steps: (1) binarization of the BOLD signal; (2) estimation of the maximum entropy model (Boltzmann distribution); (3) construction of the disconnected map and the local minimal state (basin) of energy; and (4) calculation of the dynamic index for the energy landscape. This method was originally designed to analyze fMRI data, but in principle, it is also applicable to other types of data. Based on experience, the energy landscape analysis method has a better performance when the number of variables is approximately 6 to 15. For a model with more variables, the calculation cost becomes significant, and the interpretation of the results becomes difficult. The energy landscape analysis process is shown in Figure 1.

## 3. Results

### 3.1. Construction of the triple-network energy landscape in the resting-state brain

Based on the structure of the energy landscape described above, we subsequently conducted studies on the dynamic characteristics of the triple networks in the resting-state brains of AD patients and NC subjects.

**Figure 2:**
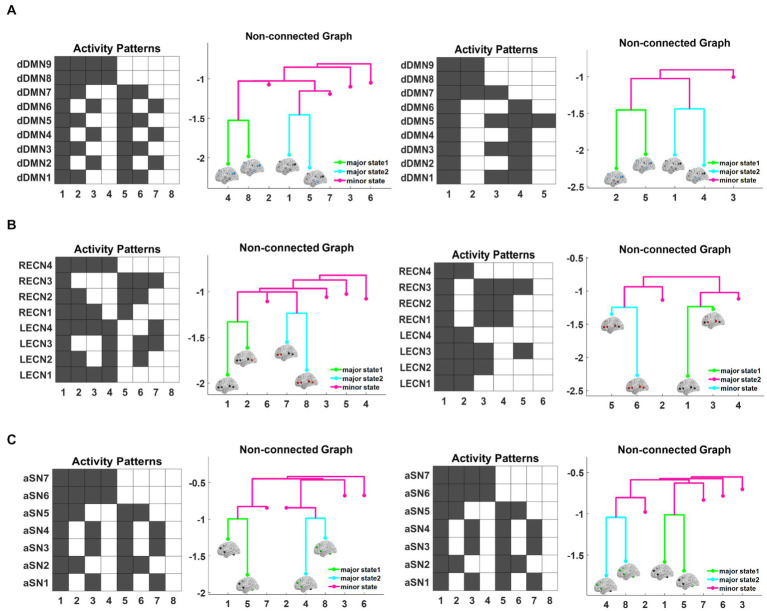
The structure of the energy landscape is shown for DMN from AD patients and NC subjects **(A1–A4)**, CEN **(B1–B4)**, and SN **(C1–C4)**. Comparing the three networks reveals that the differences between AD and NC are the least in SN and the greatest in DMN.

First, we constructed the corresponding energy landscape with fMRI data from two groups of subjects in the triple networks. The structure of the energy landscape in the triple networks from NC subjects and AD patients is shown in Figure 2. Nine ROIs were selected for the DMN, for a total of $2^{9}$ activation states. The diagram in Figure 2A1 shows the activity patterns corresponding to the 8 local minimal states in AD patients, where black represents the inactivated state and white the activated state. The figure shows strong complementarity between the brain area states of these 8 activation patterns. For example, in pattern 1, all 9 brain areas are inactive, while in pattern 8, all 9 brain areas are activated; in pattern 2, only the left and right angular gyrus and precuneus are activated, while the remaining brain areas are inactive, which is completely opposite and complementary to pattern 7. Figure 2A2 shows the nonconnected graph of the local minimal state of AD patients, which visualizes the basic characteristics of each activity pattern, and the branches of the tree represent the hierarchical structure of each activity pattern in the energy landscape. Each branch corresponds to a local minimal state, and the difference in height between each two states is the energy barrier, which measures the amount of energy required to jump between these two states. The larger the energy barrier, the less easy it is to jump between states and the lower the frequency of occurrence. Conversely, if the jump between neighboring states is easier, the frequency is higher on the time scale. As shown in Figure 2A2, pattern 4 and pattern 8, pattern 1 and pattern 5 are neighboring patterns, and their energy is relatively low, while the energy of the remaining four states is relatively high. Based on this structural feature of the energy landscape and the similarity between the two groups of subjects, we grouped the two lower energy pairs of local minimal states into two major brain states, denoted by major state 1 and major state 2, and the other higher energy local minimal states were uniformly defined as secondary brain states, which were denoted as minor states. These three states correspond to different colors in the nonconnected graph. The subsequent representation of the triple-network dynamics is based on these three states (major state 1, major state 2 and minor state) (Table 5).

**Table 5:** Brain coordinates in the SN.

Number	ROI	Brain area	BA	MNI space		
				*x*	*y*	*z*
1	aSN1	Left Middle Frontal Gyrus	10	−32	45	26
2	aSN2	Left Insula	13	−41	15	−2
3	aSN3	Left Cingulate Gyrus	32	−2	17	45
4	aSN4	Right Middle Frontal Gyrus	10	28	43	26
5	aSN5	Right Insula	13	44	13	1
6	aSN6	Left Cerebellum	/	−33	−54	−42
7	aSN7	Right Cerebellum	/	33	−55	−43

Figure 2A3 shows five activation patterns corresponding to the local minimal state in the DMN energy landscape of NC subjects, where pattern 2 is characterized by activation in the left and right angular gyrus, left and right medial frontal gyrus, cingulate gyrus and precuneus, which are complementary to pattern 4. In pattern 3, only the left and right para-hippocampal gyrus, left and right angular gyrus and precuneus brain regions are activated, while the remaining brain regions are inactive and have no complementary activation patterns. Compared with the results from the AD group, the NC group has 3 fewer brain activity patterns. Figure 2A4 shows the nonconnected graph of the energy landscape of the DMN in NC subjects with a structure similar to AD, pattern 2. Pattern 5, pattern 1 and pattern 4 are adjacent to each other, the energy required to jump between two states is smaller, and the energy of these four patterns is significantly lower than pattern 3. We inferred that these four patterns potentially represent the major activity patterns of the DMN in the resting state of NC subjects.

Figure 2B1 shows the activity patterns corresponding to the 8 local minimal states in AD patients, which are represented by the combination of 8 ROI activation states. Similar to the DMN, a strong complementary relationship exists between these 8 activation patterns corresponding to the states of brain regions. For example, in pattern 1, all 8 brain regions are inactive, which is complementary to pattern 8, where all 8 brain regions are activated; in pattern 3, only the left and right middle frontal gyrus and the left and right inferior parietal lobule are active, which is complementary to the activation status of pattern 6. Figure 2B2 shows the nonconnected graph of the local minimal state in AD patients, where pattern 7 and pattern 8, as well as pattern 1 and pattern 2 are adjacent to each other, and the energy of these four patterns is relatively low compared to other patterns. Figure 2B3 shows six activation patterns corresponding to the local minimal states in the CEN energy landscape of NC subjects. Similar to the results from AD patients, the activation patterns are complementary, such as the lack of activation in all brain regions in pattern 1 that is complementary to activation in pattern 6; in pattern 2, only the right precentral gyrus, right middle frontal gyrus and right inferior parietal lobule are activated, which is complementary to the status of pattern 4. However, compared with subjects with AD, pattern 3 and pattern 5 in NC subjects have no complementary patterns, and two patterns of brain activity are missing. The nonconnected graph in the CEN of NC subjects is shown in Figure 2B4. Similarly, patterns 5 and 6 and patterns 1 and 3 are neighboring, the energy required to jump between states with close branches is smaller, and two additional states with higher energy are observed. Overall, the structure of the CEN energy landscape for the AD and NC groups is similar to their DMN results.

Figure 2C1 shows 8 local minimal states corresponding to activation patterns in AD patients, which are represented by the combination of eight ROI activation states. All activation patterns in the figure are complementary. For example, in pattern 2, the left and right middle frontal gyrus and left cingulate gyrus are activated, while the remaining brain regions are not activated, which is complementary to the states of pattern 7. In pattern 3, only the left and right insula are activated, which is completely opposite to the state of pattern 6. Figure 2C2 shows the nonconnected graph of the local minimal states of AD patients, where pattern 1 and pattern 5, as well as pattern 4 and pattern 8, are adjacent to each other, and the energy of these four patterns is relatively low compared to the other patterns. Figure 2C3 represents the 8 brain area activation patterns in the SN energy landscape of NC, which are completely consistent with the activation patterns of AD patients. The nonconnected graphs of the SN in NC subjects are shown in Figure 2C4, with pattern 1 and pattern 5, as well as pattern 4 and pattern 8 located adjacent to each other, and the energy of these two groups of patterns is significantly lower than the other four states. We infer that these four patterns potentially represent the major activity patterns in the resting-state SN of NC subjects. Overall, unlike the DMN and CEN, the results of the energy landscape analysis of the SN network in the AD and NC groups were less different.

### 3.2. Dynamic characterization of the triple-network activity patterns in the brain

Based on the accurately fitted energy landscape model mentioned above, some frequently occurring brain major states are sufficiently representative of the resting-state brain activity pattern. The energy landscape analysis revealed the hierarchical relationships between the 2^n^ states by calculating the energy values for all possible brain activity patterns and systematically searching for brain activity patterns with the lowest local energy values that play a dominant role and are more easily observed compared to others. This value is essentially a statistical indicator of the probability that each activity pattern occurs on a time scale. Patterns of activity with lower energy values tend to occur more frequently, and these patterns are considered relatively more stable.

The results of the study showed that the energy landscape of the triple networks of subjects in the AD and NC groups had a similar structure that was dominated by the activity patterns with complementary relationships. Moreover, both had two sets of neighboring and low-energy activity patterns. Meanwhile, the remaining patterns were relatively high in energy. This suggests that the other local minimal states were less stable than these four patterns, which all had similar activation patterns in the triple networks. For instance, patterns 1/4/5/8 in the DMN of AD patients was similar to patterns 1/2/4/5 in the ROI activation pattern of NC subjects.

Based on the classification of the triple-network brain activity states described above, we selected some common dynamic indicators to quantitatively represent the dynamic characteristics of the three networks in the subjects, which include the appearance frequency, transition frequency and duration.

**Figure 3:**
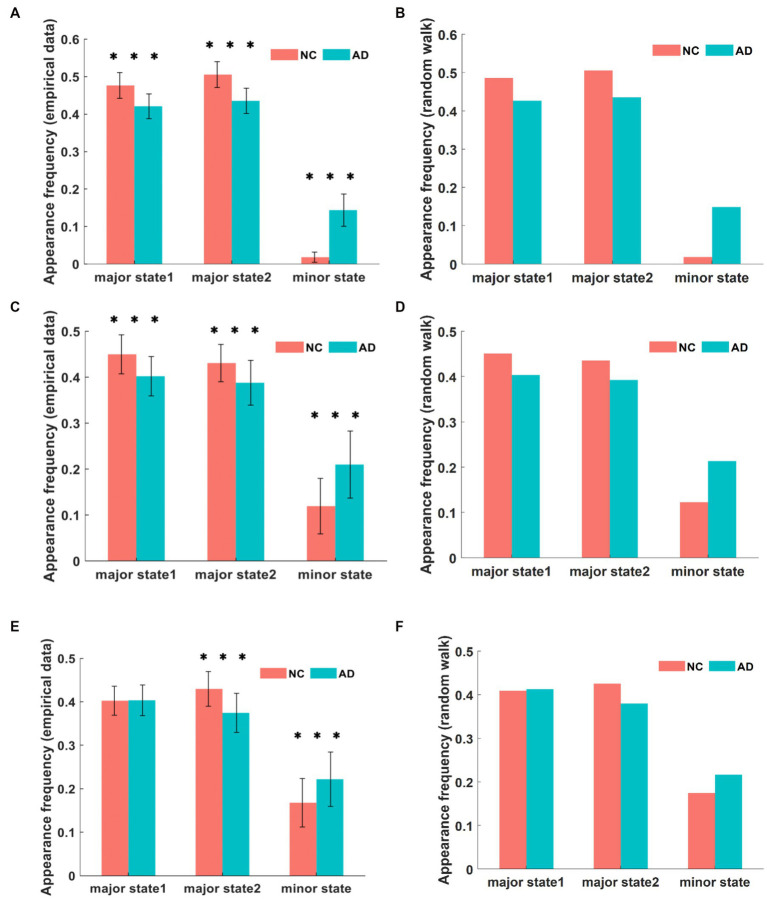
The frequency of different brain states in DMN between AD and NC in empirical data is compared in **(A)**. In simulation, the frequency of appearance for each state in DMN is shown in **(B)**. The frequency of appearance of each state in CEN is presented in **(C,D)** for empirical data and simulation, respectively. Similarly, the frequency of appearance of each state in SN is shown in **(E,F)** for empirical data and simulation. Although no significant difference is observed between the two types of data, there are significant differences between AD and NC, and these differences have varying degrees in the three networks. ***p < 0.001 in t-test.

First, we calculated the frequency of occurrence of each state in the fMRI time series and used it to quantify the dominance of different states. The comparison of the frequency of each state in the DMN between the AD and NC groups is shown in Figure 3A. Within a single group of subjects, the frequencies of major state 1 and major state 2 were much higher than that of the minor state. The proportion of the frequency of brain states in the DMN of AD patients was similar to that of NC subjects, and both showed a significantly higher proportion of major states than minor states. This finding is consistent with the results of the energy landscape analysis, where states with low energy appear more frequently in time, are more stable, and dominate the pattern of brain activity. In addition, for major state 1, the frequency of occurrence was 42.1% in AD patients and 47.6% in NC subjects, representing a significantly lower percentage in AD patients than in NC subjects (*p* < 10^−8^). Similarly, the frequency of major state 2 was 43.5% in AD patients and 50.5% in NC subjects, which was significantly lower in AD patients than in NC subjects (*p* < 10^−12^). However, the frequency of the minor state in the AD group was 14.4%, while the frequency in the NC group was 1.8%, representing a significantly higher frequency in the AD group than in the NC group (*p* < 10^−25^).

Figure 3C shows the comparison of the frequency of each state in the CEN between the AD and NC groups. Within a single group of subjects, the proportions of the frequencies of major state 1 and major state 2 were significantly higher than that of the minor state. The proportions of the frequencies of CEN brain states in AD patients was similar to that in NCs, both showing a significantly higher proportion of major states than minor states. Similarly, for major state 1, the frequency of occurrence was 40.2% in AD patients and 44.9% in NC subjects, and was significantly lower in AD patients than in NC subjects (*p* < 10^−4^). The frequency of major state 2 was 38.8% in AD patients compared with 43.1% in NC subjects, and was significantly lower in AD patients than in NC subjects (*p* < 10^−3^). In addition, the frequency of the minor state was 21.1% in the AD group compared with 11.9% in the NC group, representing a significantly higher frequency in the AD group than in the NC group (p < 10^−6^). The results of this analysis were generally similar to those obtained for the DMN network.

Figure 3E shows the frequency results for each state in the SN between the AD and NC groups. Within a single group of subjects, the frequency proportions of major state 1 and major state 2 were significantly higher than those of the minor state. For different groups of subjects, a significant difference was not observed between groups in the frequency of major state 1, which was different from the results obtained for the first two networks (p = 0.9026). In addition, the frequency of major state 2 was significantly lower in patients with AD than in NC subjects (p < 10^−6^), and the frequency of major state 2 was 37.5% in patients with AD and 42.9% in NC subjects. In contrast, the frequency of minor states was significantly higher in AD patients than in NCs (p < 10^−3^), as the frequency of minor states was 22.2% in AD patients and 16.8% in NC subjects.

**Figure 4:**
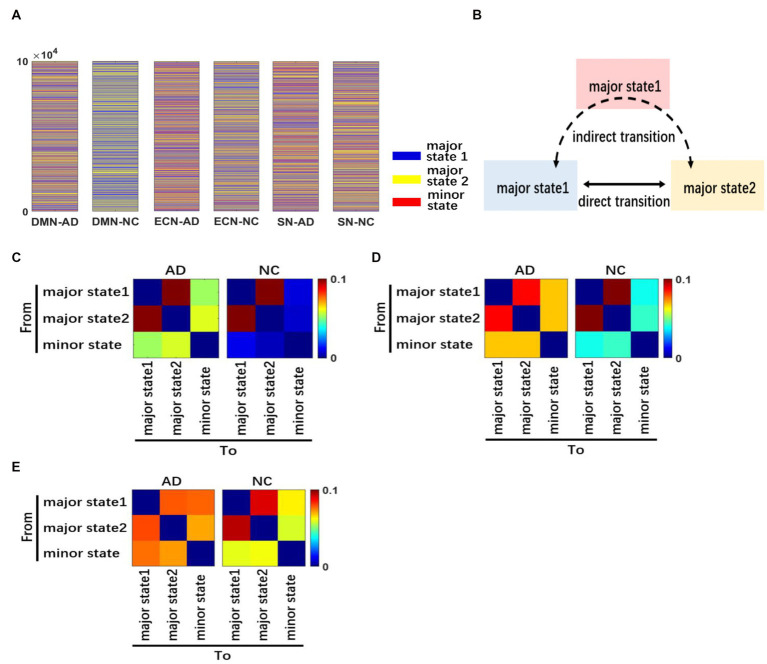
Dynamics of brain activity patterns. We performed a $10^{5}$ random-walk numerical simulation to characterize the dynamics in brain states for each network of AD and NC **(A)**. A schematic diagram showing different types of transitions between brain activity states is presented. We divide the transition pattern of major states into direct and indirect forms **(B)**. The transition frequency matrix between different brain states of DMN **(C)**. The transition frequency matrix in CEN **(D)**. The transition frequency matrix in SN **(E)**. The three networks have different degrees of difference.

The aforementioned results indicate that the frequency of major brain states in AD patients is significantly lower than that in NC subjects, while the frequency of minor states is significantly higher than that in NC subjects. We further analyzed the dynamics of brain activity states by calculating and visualizing the transition frequency matrices between different brain activity states in the triple networks of the AD and NC groups. The transition frequency matrix of the DMN is shown in Figure 4C, and it depicts that the activity pattern transitions in both the AD and NC groups are mainly concentrated between the two major states. The difference is that the transition frequency between the minor state and the major state is higher in patients with AD, while it is approximately 0 in NC subjects.

The transition frequency matrix between different brain states of the CEN is shown in Figure 4D, and it indicates that although the activity state transitions in both the AD and NC groups were mainly concentrated between the two major states, the concentration trend was more obvious in NC subjects. Meanwhile, the transition frequency between the minor state and major states was significantly higher in AD patients.

The transition frequency matrix between different brain states of the SN network is shown in Figure 4E. Compared with the first two networks, the transition frequency between the minor state and major states was significantly increased for both groups. However, the active state transitions of NC subjects were still mainly concentrated between the two major states, while patients with AD had similar transition frequencies between the minor state and major states compared to the two major states.

In summary, the results showed that the minor state was less frequent in both AD patients and NC subjects, and the transition frequency from the minor state was also low. Therefore, we classified the transitions of brain activity states into the following two types: direct transitions between major states only and indirect transitions between the two major states through the minor state. For simplicity, we refer to the minor state as the intermediate state between the major states, which mainly appears in the jump transition between major states. The two types of state transitions are defined in Figure 4B.

**Figure 5:**
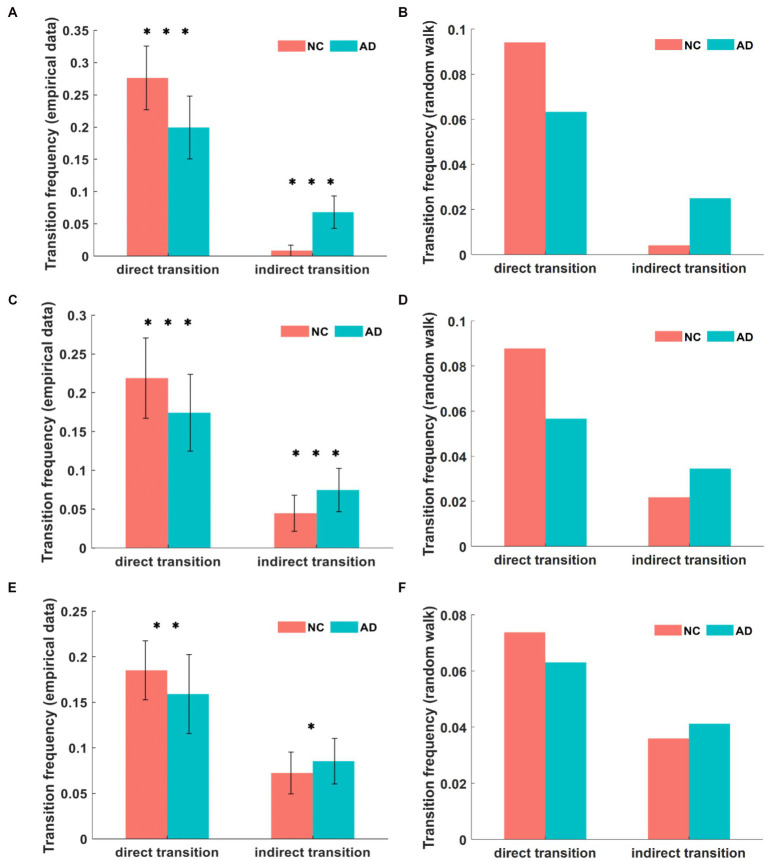
In the empirical data, the comparison of transition frequencies between major states in DMN **(A)**. In the simulation, the transition frequencies in DMN **(B)**. The transition frequencies between major states in CEN from empirical data and simulation **(C,D)**. The transition frequencies in SN from empirical data and simulation **(E,F)**. We can find that the empirical value is greater than the simulation value, and there are different degrees of variability in AD and NC among the three networks. *p < 0.05, **p < 0.01, ***p < 0.001 in t-test.

Based on this definition, we further calculated the frequency of direct transitions between major states and the frequency of indirect transitions between major states through minor states and compared the statistical analysis of AD patients with NC subjects. The comparison of the transition frequencies between major states in the DMN for both groups is shown in Figure 5A. The direct transition frequencies were higher than the indirect transition frequencies in both the AD and NC groups. However, the direct transition frequency between major states was significantly lower in AD patients than in NC subjects (*p* < 10^−8^), being 19.9% in AD patients and 27.6% in NC subjects. In contrast, the indirect transition frequency for major states was significantly higher in subjects with AD than in NC subjects (*p* < 10^−21^), being 6.8% in the AD group compared to 0.9% in the NC group.

The comparison of the transition frequencies between major states in the CEN for both groups is shown in Figure 5C. The direct transition frequency of major states in the CEN was 17.4% in AD patients and 21.9% in NC subjects, which was significantly lower in AD patients than in NC subjects (*p* < 10^−3^). Conversely, the frequency of indirect transitions between major states was 7.5% in patients with AD compared to 4.4% in NC subjects, and the value of the AD group was significantly higher than that for the NC group (*p* < 10^−5^).

The comparison of the transition frequencies between major states in the SN for both groups is shown in Figure 5E. The direct transition frequency of the SN was 15.9% in AD patients and 18.5% in NC subjects, indicating a significantly lower value for AD patients than for NC subjects (*p* = 0.0048). In contrast, the indirect transition frequency of the major state was 8.5% in the AD group compared to 7.2% in the NC group, where the value of the AD group was higher than that of the NC group (*p* = 0.025).

**Figure 6:**
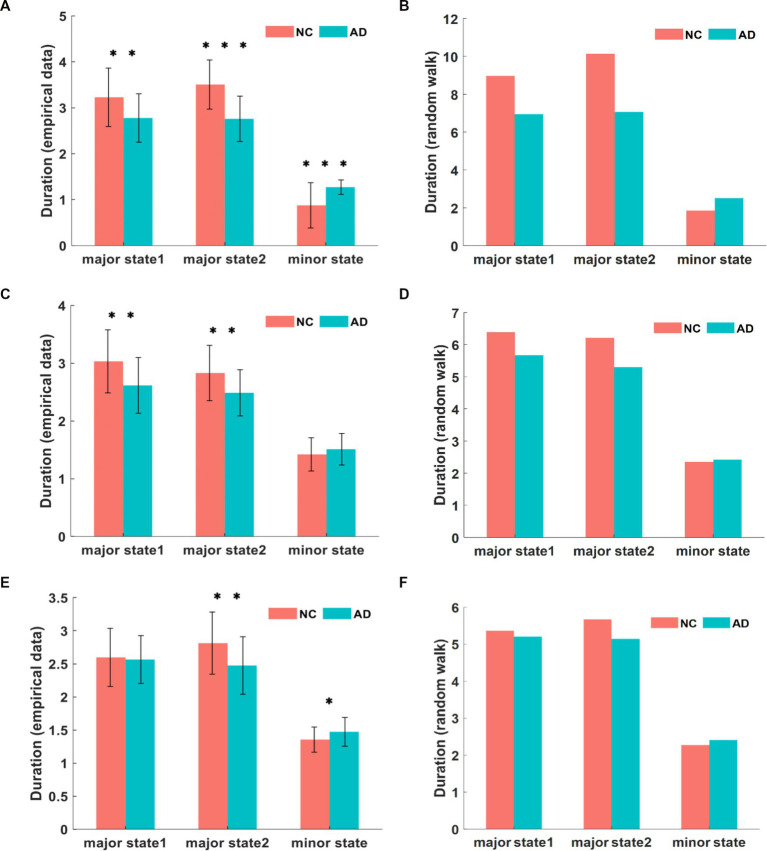
The comparison of empirical data on state duration in DMN **(A)**. The comparison of simulated data for state duration in DMN **(B)**. The state duration of each state in CEN from empirical data and simulation **(C,D)**. The state duration in SN from empirical data and simulation **(E,F)**. It can be found that the empirical value is smaller than the simulated value, and there are different degrees of variability in AD and NC among the three networks. *p < 0.05, **p < 0.01, ***p < 0.001 in t-test.

The state transition frequency difference in the three networks was similar in both groups, with lower direct transition frequencies and higher indirect transition frequencies in AD patients compared to NC subjects. However, within the AD group, the direct transition frequencies were significantly higher than the indirect transition frequencies. This abnormal state transition indicates that the brain activity states of AD patients tend to remain in minor states for a longer time than those of NC subjects. We confirmed this inference by subsequently calculating the duration of brain activity patterns in the three states, where this index was the average duration of each state over the time series, quantified as the mean length of repetition over time. The unit of duration is step of TR, shortened to s. The comparison of the mean duration in the DMN between both groups is shown in Figure 6A. The duration of both major states in the AD group was significantly smaller than that of the NC group, where the mean duration of major state 1 was 2.78 s in patients with AD and 3.23 s in NC subjects (*p* = 0.0019), and the mean duration of major state 2 was 2.76 s in patients with AD and 3.51 s in NC subjects (*p* < 10^−8^). However, the duration of the minor state in AD patients was significantly longer than that in NC subjects, with a mean duration of 1.27 s in AD patients and 0.88 s in NC subjects (p < 10^−4^). The comparison of the mean duration in the CEN between both groups is shown in Figure 6C. The duration of both major states in the AD group was significantly smaller than that in the NC group, where the average duration of major state 1 in patients with AD was 2.62 s and 3.03 s in NC subjects (*p* = 0.0011); the average duration of major state 2 in the AD group was 2.49 s and 2.83 s in the NC group (*p* = 0.0017). Unlike the results for the DMN, the duration of the minor state in the CEN of AD patients was not significantly different from that of NC subjects, with a mean duration of 1.51 s in AD patients and 1.42 s in NC subjects (*p* = 0.18). Figure 6E shows the comparison of the mean duration of states in the SN between both groups. The mean duration of major state 1 was 2.56 s in AD patients and 2.59 s in NC subjects, which were not significantly different (*p* = 0.74); the mean duration of major state 2 was 2.47 s in AD patients and 2.81 s in NC subjects, and was significantly smaller in the AD group than in the NC group (*p* = 0.0024). In addition, the duration of the minor state in AD patients was longer than that in NC subjects (*p* = 0.017), and the average duration of the minor state was 1.47 s in AD patients and 1.35 s in NC subjects.

Compared to the NC group, brain activity in the AD group was characterized by a shorter duration in major states and a significantly longer duration in transition states, but the duration of major states was significantly longer than that of transition states within the AD group. Notably, the duration of each state in the triple networks differed to varying degrees, with the DMN differing most significantly, followed by the CEN and SN.

In conclusion, compared with the NC group, the AD group has a higher frequency and duration of minor states and a higher frequency of indirect transitions, which indirectly reflects the abnormal dynamic characteristics of brain activity states in individuals with AD.

### 3.3. Random-walk simulation of the dynamics of the energy landscape

We performed $10^{5}$ random walk data simulations to characterize the dynamic changes in the brain states of the AD and NC groups, as shown in Figure 4A. The variability of dynamic changes in brain states differed among the three networks, with the most significant difference noted in the DMN, followed by the CEN, and the least significant difference detected in the SN. Compared with patients with AD, the frequency of major states in the DMN of NC subjects was extremely high, but the frequency of minor states was low. In the CEN, the frequency of minor states in NC subjects is increased, but there was still a gap compared with AD patients. In the SN, the difference between the NC and AD groups was significantly reduced, and the frequency of each state was similar. The SN usually plays a moderating and transitional role between the DMN and CEN; thus, we hypothesized that the coordination relationship between the networks of dynamic brain systems in patients with AD might be less affected by the disease.

We characterized the dynamics of three core networks related to cognitive functions in AD and NC groups based on the energy landscape. Based on the results, the energy landscape structures of the AD and NC groups are similar, the tree-like branching structures of the nonconnected graph have two sets of adjacent and relatively low energy local minimal states, and some similarity in the activation patterns of the three networks is observed between the two groups. Nevertheless, the results of the energy landscape analysis and the differences in dynamic characteristics of the AD and NC groups were significant. We analyzed three dynamic characteristics. The first characteristic is the appearance frequencies of different brain states, as shown in Figure 3B, Figure 5B and Figure 6B. In both the AD and NC groups, all three networks showed a significantly higher frequency of major states over time than minor states, while the frequency of major states in patients with AD was lower than that in NC subjects, which was also evident from the transition frequency matrix between the different brain activity states. Thus, the activity of minor states in patients with AD increased and the total number of appearances of major states decreased significantly in the same time period. The second feature is the frequency of different transition modes of the major state, as shown in Figure 3D, Figure 5D and Figure 6D. The direct transition frequencies between the major states in patients with AD are all lower than those of NC subjects. In contrast, their indirect transition frequencies are significantly higher than NC subjects. Therefore, the minor state of patients with AD, which functions as a transition state, affects the stability of the switching between the major states, and thus the direct switching between the major states is more easily interrupted by the appearance of the minor states. Finally, we analyzed the average durations of the three networks in the AD and NC groups, and the results in Figure 3F, Figure 5F and Figure 6F showed that the average durations of the two major states in patients with AD were less than those in NC subjects. Combining the results for these three characteristics, we propose that the major states in patients with AD showed instability phenomena, which might arise from the abnormal minor states. The total number of minor state appearances increased on the time scale, the AD group was more likely to jump from the major state to the minor state, and the duration of each minor state appearance increased. Based on these results, the brain regions associated with the minor state are more likely to be activated, and the activation level is increased and more persistent, disrupting the original steady state.

### 3.4. Study of the correlation between triple-network dynamic characteristics and clinical MMSE scores

We further explored whether the abnormal dynamic characteristics of the triple-network brain activity states in patients with AD are related to the disease by analyzing the dynamic indicators in combination with the subjects’ clinical behavioral data, the scores of which range from 0–30 points, with lower scores representing a more severe intellectual impairment. In the figure, R represents the correlation coefficient, and P represents the level of significance of the correlation.

**Figure 7:**
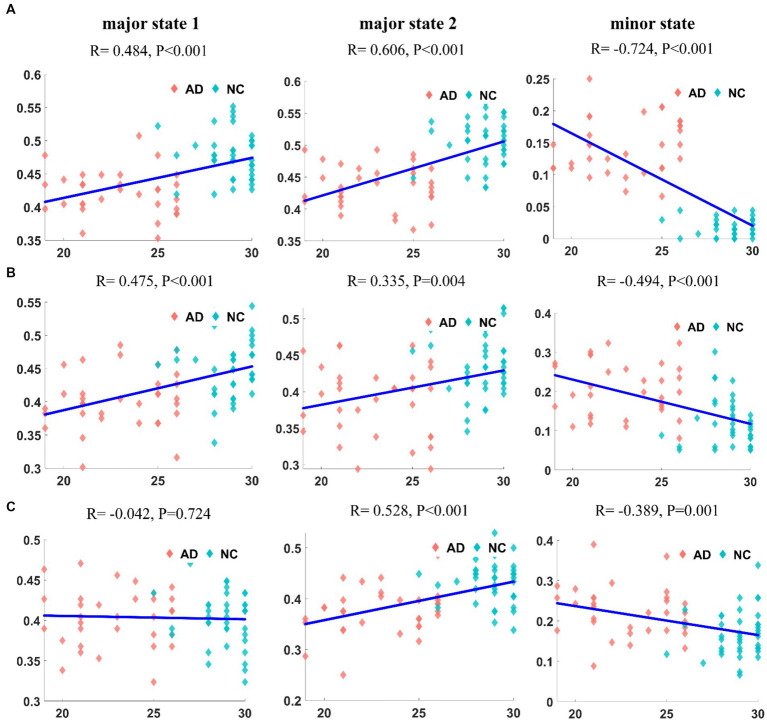
The correlation between the frequency of each state and MMSE in DMN **(A1–A3)**. The correlation between the frequency of each state and MMSE in CEN **(B1–B3)**. The correlation between the frequency of each state and MMSE in SN **(C1–C3)**. It can be found that the frequency of appearance and MMSE are correlated, and the correlation is most significant in DMN.

We first calculated the correlation between the appearance frequencies of each state in patients with AD and the MMSE scores. The results for the DMN are shown in Figure 7A. The frequency of appearance of both major states had a significant positive correlation with the MMSE score (*R* = 0.484, *p* < 10^−4^; *R* = 0.606, *p* < 10^−7^). In contrast, the frequency of the minor state was significantly negatively correlated with MMSE scores (*R* = − 0.724, *p* < 10^−12^). The correlation between the frequency of each state in the CEN of patients with AD and the MMSE score is shown in Figure 7B. The frequencies of both major states were positively correlated with the MMSE score (*R* = 0.475, *p* < 10^−4^; *R* = 0.335, *p* = 0.004). In contrast, the frequency of the appearance of the minor state was significantly negatively correlated with the MMSE score (*R* = −0.494, *p* < 10^−4^). The correlation between the frequency of each state in the SN of AD patients and MMSE scores is shown in Figure 7C. No correlation was observed between the frequency of major state 1 and the MMSE score (*R* = 0.042, *p* = 0.724), but a significant positive correlation was observed between the frequency of major state 2 and the MMSE score (*R* = 0.528, *p* < 10^−5^). In contrast, the frequency of the minor state was negatively correlated with the MMSE score (*R* = −0.389, *p* = 0.001).

**Figure 8:**
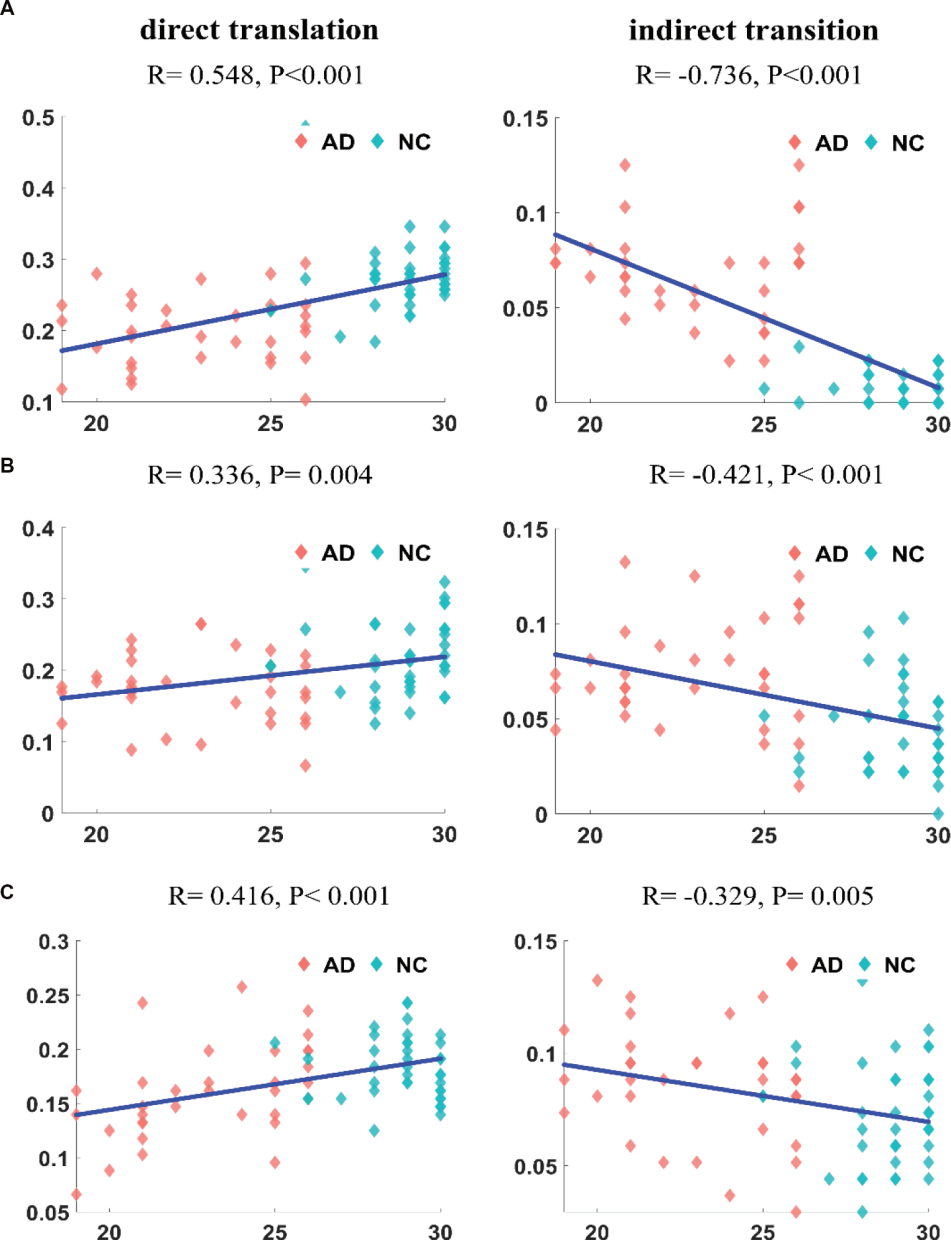
The correlation of transition frequencies between major states and MMSE in DMN **(A1,A2)**. The correlation of transition frequencies between major states and MMSE in CEN **(B1,B2)**. The correlation of transition frequencies between major states and MMSE in SN **(C1,C2)**. It can be found that the frequencies of different transition methods are significantly correlated with MMSE, and the degree of correlation is different for the three networks.

Second, we calculated the correlation between the transition frequency of patients with AD and the MMSE score. The result for the DMN is shown in Figure 8A. The direct transition frequency was significantly positively correlated with the MMSE score (*R* = 0.548, *p* < 10^−6^). In contrast, a significant negative correlation was detected between the indirect transition frequency and MMSE score (*R* = –0.736, *p* < 10^−12^). The correlation between the frequency of transitions in the CEN and the MMSE score is shown in Figure 8B. The frequency of the direct transition in patients with AD was positively correlated with the MMSE score (*R* = 0.336, *p* = 0.004). In contrast, the indirect transition frequency had a negative correlation with the MMSE score (*R* = −0.421, p < 10^−3^). The correlation analysis between the frequency of transitions in the SN and the MMSE score is shown in Figure 8C. The frequency of the direct transition in patients with AD was positively correlated with the MMSE score (*R* = 0.416, *p* < 10^−3^). The indirect transition frequency, on the other hand, had a negative correlation with the MMSE score (*R* = −0.329, *p* = 0.005). The correlations between each transition frequency and the MMSE score were similar for the three networks.

**Figure 9:**
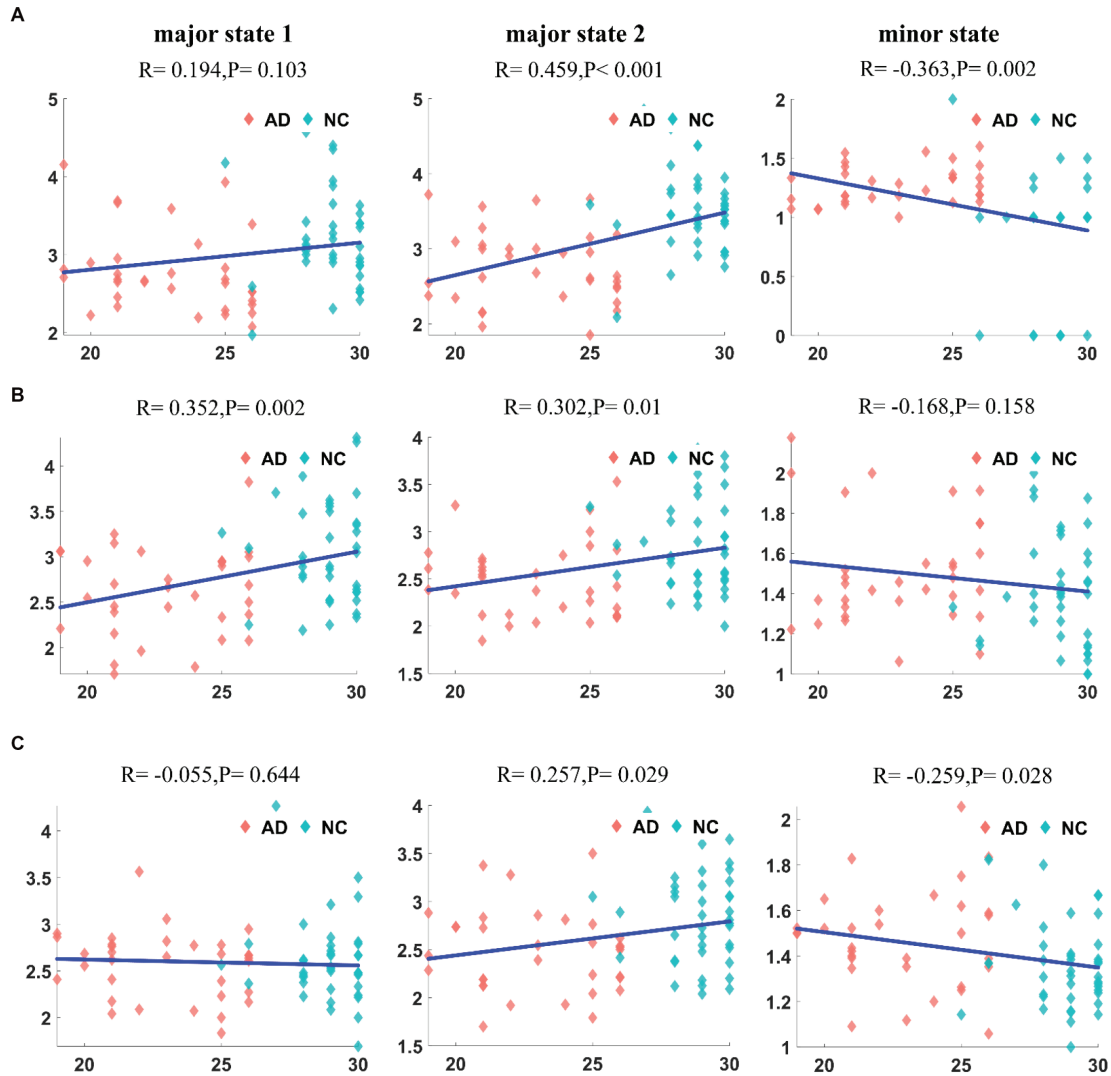
The correlation between the duration of each state and MMSE in DMN **(A1–A3)**. The correlation between the duration of each state and MMSE in CEN **(B1–B3)**. The correlation between the duration of each state and MMSE in SN **(C1–C3)**. It can be found that there is a significant correlation between duration and MMSE, but the degree of correlation is not significant in SN.

Finally, we analyzed the correlation between the mean duration of states in patients with AD and the MMSE score, and the results for the DMN are shown in Figure 9A. The correlation between the mean duration of major state 1 and the MMSE score was not significant (*R* = 0.194, *p* = 0.103), but the mean duration of major state 2 displayed a significant positive correlation with the MMSE score (*R* = 0.459, *p* < 10^−4^). In contrast, the mean duration of the minor state was significantly negatively correlated with the MMSE score (*R* = −0.363, *p* = 0.002). Notably, due to the low frequency of the minor state in patients with AD, some outliers in their mean duration (duration close to 0) are present, which may have affected the results of the correlation analysis. The correlation analysis between the duration in the CEN of patients with AD and the MMSE score is shown in Figure 9B. Unlike the previous results, the mean durations of both major states in the CEN were positively correlated with the MMSE score (*R* = 0.352, *p* = 0.002; *R* = 0.302, *p* = 0.01). A weaker negative correlation was observed between the duration of the minor state and MMSE score (*R* = −0.168, *p* = 0.158). The correlation analysis between the mean duration in the SN of patients with AD and the MMSE score is shown in Figure 9C. The mean duration of major state 1 in patients with AD was not correlated with the MMSE score (*R* = −0.055, *p* = 0.644), whereas the mean duration of major state 2 was weakly positively correlated with the MMSE score (*R* = 0.257, *p* = 0.029). In addition, a weaker negative correlation was observed between the duration of the minor state and the MMSE score (*R* = −0.259, *p* = 0.028).

## 4. Discussion

### 4.1. Time-varying characteristics of the triple-network dynamics in the resting brain

In the present study, we explored the differences in dynamic characteristics of three networks in the resting brain between the AD and NC groups by conducting an energy landscape analysis. In all three networks, the brain activity patterns of patients with AD lacked a more stable major state compared to NC subjects, while the minor state was more active. This property may cause frequent switching in resting-state brain activity patterns between different substates, increasing the frequency of transitions between major and minor brain states, and the occurrence of minor states is significantly higher. Patients with AD experience difficulty in focusing on the major state for a long time and lose stability of the three networks.

According to previous studies of the normal ageing process of the brain, its internal balance system actively inhibits the effects of various genetic and environmental factors, whereas the balance system fails in patients with neurodegenerative diseases such as AD. The instability of spontaneous neuronal activity in cortical and hippocampal circuits is a typical feature of AD (Palop et al., 2007; Frere and Slutsky, 2018). Researchers observed a strong correlation between the formation of amyloid plaques, the appearance of overactive neurons and the impairment of learning abilities in animals. By performing functional studies of individual cortical neurons in a mouse model of AD, researchers found that the presence of overactive neurons near amyloid plaques accounted for 50% of neurons, which was 16 times more than that in normal mice. This phenomenon causes serious dysfunction of the neural network of AD mice (Busche et al., 2008). Impaired synaptic function and decreased neural plasticity are the early symptoms of AD and are closely related to the decline in cognitive ability. Based on a study using transcranial magnetic stimulation, cortical inhibition is weakened in AD patients, and the cortical excitability is significantly higher than that of elderly individuals with normal cognition. Lower cognitive performance is significantly associated with higher cortical excitability and lower inhibition (Spires-Jones and Knafo, 2012; Chou et al., 2022). These results are consistent with the findings from our study that the stability of major states in the network associated with cognitive function is disrupted in patients with AD compared with normal subjects, and the patient’s brain is unable to easily suppress this active abnormal activity. In recent years, Ma et al. have used co-activation pattern analysis to study the AD brain and found that the increase in transition and CAP entropies and the diversity of CAP transition probabilities suggest variable information flow and higher system uncertainty Ma et al. (2020). Our study has also revealed that the triple-networks of AD patients exhibit decreased main state occurrence, increased minor state occurrence, and increased system instability, which is consistent with the previous study. Sendi et al. observed during normal brain development toward very mild AD that there were significant interruptions in all states, and that connectivity of multiple networks, such as the subcortical, auditory, and visual networks, decreased (Sendi et al., 2021). Recombination patterns were also found in connections within and between multiple networks. Similarly, our results show changes in the energy landscape of different states in AD patients when compared to those of NC individuals. This suggests a network reorganization may be occurring in AD patients. Ghanbari et al. employed a sliding window method to estimate the dynamic functional connectivity (dFC) of each network, from which they extracted the Mean of Redundancy (MOR) and Fluctuation of Redundancy (FOR) features. Statistical analysis based on these features revealed that redundancy significantly increased in AD patients compared to NC individuals (Ghanbari et al., 2023). Our study also found that the stability of the main state decreased, while the indirect switching increased in AD patients, which may account for the increase in redundant dynamic characteristics.

Therefore, we considered that all three core networks associated with cognitive function are affected by AD-related disorders. Previous studies have observed varying degrees of damage to the three networks in AD patients, where the number of functional connections in the module is significantly reduced and closely related to cognitive impairment.

The DMN consists mainly of the posterior cingulate cortex (PCC), precuneus (PCu), and inferior parietal lobe (IPL) and is mainly associated with some higher cognitive functions of the brain, such as situational memory, visuospatial processing, and consciousness (Ciftci, 2011; Mohan et al., 2016). The DMN in the normal brain is characterized by a symmetrical, well-organized pattern (Raichle et al., 2001). However, in the mild AD group, the symmetry or functional connectivity between interhemispheric homogeneous regions decreases in some disease-related functional networks. Patients with AD perform normally when using intrahemispheric processing but poorly when interhemispheric communication is needed. This loss of symmetry may reflect a state of cognitive decline and imbalance in the functional networks of the patient’s brain (Lee et al., 2009; Chen et al., 2013). For example, the functional connectivity of the precuneus, posterior cingulate and medial frontal cortex in the DMN of patients with AD is reduced, and the correlation between nodes in the DMN is reduced (Palesi et al., 2016). The CEN is the functional brain network associated with executive tasks, mainly comprising the dorsolateral prefrontal cortex (DLPFC) and the posterior parietal cortex (PPC), covering multiple medial-frontal regions, including the anterior and paracallosal cingulate, which are presumed to help process information related to working memory, decision-making and retention, and operational target detection (Ramezanzadeh et al., 2014; Wang et al., 2015). Compared to NC subjects, patients with AD exhibit enhanced functional connectivity in the suprafrontal gyrus (SFGmed.L) and middle frontal gyrus (MFG.L) regions of the CEN network, while the anterior cingulate cortex (ACG.R) region of the CEN shows reduced functional connectivity. These changes may contribute to impaired executive function (Jilka et al., 2014). The SN is similar to a “dynamic switch” network that plays a regulatory and transitional role, mainly comprising the ventral lateral prefrontal cortex (VLPFC) and the anterior cingulate cortex (ACC), and is proposed to play a key coordinating role between the CEN and the DMN (Ciftci, 2011; Brier et al., 2012). Functional connectivity within the SN is not significantly different between the NC and AD groups, but abnormal connectivity with the DMN and CEN occurs in AD patients (Wang et al., 2015).

However, among the three networks, the dynamic characteristics of the DMN and CEN were significantly different between the two groups compared with the SN. For example, the intermediate transition states of the DMN and CEN in the AD group are greater than those in the NC group, whereas the energy landscape constructed by the SN had high similarity, corresponding to the same pattern of brain area activation. In addition, group differences in the frequency and duration of major state 1 were not found in the SN, and the differences in the probability of transitions between the major states were not as significant as in the other two networks. Our results indicated that the SN may be less affected by the disease, while the DMN and CEN are more severely damaged than the SN in the early stage of AD.

In clinical practice, AD usually begins with a situational memory impairment followed by a slow progression to wider impairments in daily activities such as attention, executive functioning, language, and visuospatial functioning, eventually leading to loss of independent daily living abilities (Dai et al., 2015). As mentioned above, the DMN plays a key role in cognitive processes, particularly in situational memory processing. Therefore, the DMN plays a central role in brain activity and connects other participating networks, indicating that AD pathology may spread from the DMN to nearby networks, including those involved in visual space and executive function, as well as other peripheral networks (Fair et al., 2008; Liu et al., 2018). Significant degeneration of functional connectivity has been observed within the DMN network in AD patients, with the bilateral angular gyrus (AG) identified as one of the typical areas. Additionally, both clusters of the right middle frontal gyrus and the superior frontal gyrus in the CEN related to the control of executive functions showed a significant decrease in functional connectivity (Sridharan et al., 2008). In contrast, no significant differences in the functional connectivity of internal networks were observed in the SN of both the AD and NC groups (Zhu et al., 2016). Compared with age-matched controls, individuals with early-onset AD showed lower functional connectivity in all networks, such as auditory, sensorimotor, and default mode networks, whereas patients with late-onset AD showed lower functional connectivity only in the DMN. Patients with early-onset AD have more extensive disorganization of brain function than those with late-onset AD (Hodges, 2006; Adriaanse et al., 2014). These results support the hypothesis that the DMN is more severely impaired than the CEN and that the SN is less affected by the disease in AD patients.

### 4.2. Comparison of the dynamic characteristics based on random walk data simulations

We conducted $10^{5}$random walk data simulations to characterize the dynamic changes in resting brain activity in the three networks of AD patients and NC subjects as an approach to verify our results. We analyzed the dynamic characteristics of the simulation data and compared them with the empirical data to verify the effectiveness and rationality of the energy landscape model. Through comparison, we found that the statistical results of the dynamic characteristics of the simulated data and empirical data were consistent. Based on the findings described above, we further confirmed that the SN is less affected by the disease. We verified that the energy landscape achieves a better description of the nonequilibrium process of switching between resting-state activity patterns in the brain, from which the stability and interactions of the states can be determined and the dynamics characteristics can be described in more detail. The method is also suitable for analyzing specific networks and ROI brain regions.

### 4.3. The relationship between the dynamic changes in the three networks and a cognitive index

We tested the correlation between the dynamic features that were significantly different in AD patients and the clinical index. The frequency of occurrence, frequency of state transitions and duration of the major states in the three networks of these subjects were correlated with the MMSE scores. The characteristics of major states were generally positively correlated with MMSE scores; the higher the score, the more stable the major states were. Meanwhile, the characteristics of minor states were negatively correlated with MMSE scores; the lower the score, the more active the minor states were. The degree of correlation differed among the three networks, with the DMN exhibiting the highest correlation with MMSE scores, followed by the CEN, and the weakest correlation was observed with the SN. Therefore, we suggest that the abnormalities in the dynamic characteristics of the three networks of patients with AD are related to their cognitive impairment, with the DMN and CEN identified as more strongly associated with cognitive impairment and the SN showing a weaker correlation.

Our research provides empirical evidence that AD patients are characterized by abnormally nonequilibrium large-scale brain network dynamics. Although previous neuroimaging studies have reported neural synchronization disorders in patients with AD and identified a unique variety of structural and functional whole-brain architectures, most studies have not directly studied brain dynamics (Uhlhaas and Singer, 2006). In contrast, we illustrate the time-varying characteristics of dynamic brain activity patterns in three core brain networks associated with cognitive function and directly report the link between abnormalities in nonequilibrium brain dynamics in patients with AD and their clinical cognitive performance. Given the results of previous studies, our current study may be considered as providing additional empirical support, emphasizing the importance of studying brain dynamics to obtain a biological understanding of various developmental and psychiatric disorders and provide a deeper understanding of the intrinsic neural mechanism and system dynamics characteristics of the AD brain in the resting state.

## 5. Limitations and future work

We have studied and revealed the dynamic characteristics of functional network activity patterns in the resting brains of AD patients from the perspective of nonequilibrium dynamics. The data used in this study are derived from subjects included in a single database, and the number of samples used in this study is limited, including only 33 AD patients and 39 healthy subjects. In the future, other data sources and a larger sample size must be considered, as well as the inclusion of patients with mild cognitive impairment as an intermediate control group, which will help to improve the reliability of the research methods and results. Additionally, the analysis performed in this study only used one clinical behavioral data point, the MMSE score of the subjects, and thus the study lacked comprehensiveness. Thus, we need to include more clinical behavioral data as indicators for the correlational analysis to increase the persuasiveness of the findings and conclusions. Currently, various artificial intelligence (AI) techniques and advanced signal processing methods have been used for accurate diagnosis of mental illnesses such as AD, Schizophrenia (SZ), and ASD (Khodatars et al., 2021; Sadeghi et al., 2022; Illakiya and Karthik, 2023). Combining dynamic indicators with deep learning can significantly reduce network training costs, which has become one of the hotspots in current research on mental illness diagnosis. We will continue to develop function magnetic resonance data analysis methods based on non-equilibrium dynamics in the future, and combine them with deep learning to strive for breakthroughs in the diagnosis of multiple neurological diseases such as AD and ASD.

## 6. Conclusion

The main focus of this paper was to perform an energy landscape analysis of three networks in the brains of patients with AD and NC subjects, to further characterize dynamics-related features based on the constructed energy landscape, and to observe the correlation between a series of dynamic characteristics and the clinical cognitive function of the subjects. In the Introduction section, we introduced the triple-network model and related background information, followed by the Methods, which described the extraction of the ROI time series and the most important method of the energy landscape analysis. Our study was divided into four parts. First, we compared the structure of the triple-network energy landscape between the two groups and then further explored the resting-state brain dynamic characteristics of the AD and NC groups. We confirmed our inference that AD brain activity patterns are in an abnormal nonequilibrium state, and the dynamics of patients with AD tend to be unstable, with an unusually high flexibility in switching between states. Then, we correlated the subjects’ dynamic features with clinical data and found that the atypical balance of large-scale brain systems in patients with AD is associated with abnormally active brain dynamics, which may explain the general cognitive impairment of patients. Finally, we simulated the dynamic changes in activity patterns using a random walk model and verified that the energy landscape analysis method can reveal the kinetic features in the model. The results of this paper are helpful for further understanding the intrinsic dynamic characteristics and pathological mechanism of the resting-state brain in AD patients.

## Data availability statement

The original contributions presented in the study are included in the article/supplementary material, further inquiries can be directed to the corresponding author.

## Author contributions

YL, CS, NY, and Z-GH designed the project. SA, TZ, SZ, CL, JJ, and YM analyzed the data. YL, SA, NY, and Z-GH interpreted the results and wrote the manuscript. All authors participated in the revision of the manuscript, read and approved the submitted version. All authors provide approval for publication of the content and agree to be accountable for all aspects of the work in ensuring that questions related to the accuracy or integrity of any part of the work are appropriately investigated and resolved.

## Funding

The work was supported by the National Key R&D Program of China (nos. 2022ZD0208500 and 2021ZD0201300), Natural Science Basic Research Plan in Shaanxi Province of China (No. 2023-JC-YB-071), Scientific Research Program Funded by Shaanxi Provincial Education Department (No. 22JP053), NNSF of China (no. 11975178), the Open Project of State Key Laboratory of Cognitive Neuroscience and Learning (no. CNLYB1802), K. C. Wong Education Foundation.

## Conflict of interest

The authors declare that the research was conducted in the absence of any commercial or financial relationships that could be construed as a potential conflict of interest.

## Publisher’s note

All claims expressed in this article are solely those of the authors and do not necessarily represent those of their affiliated organizations, or those of the publisher, the editors and the reviewers. Any product that may be evaluated in this article, or claim that may be made by its manufacturer, is not guaranteed or endorsed by the publisher.

## Footnotes

^1^ https://adni.loni.usc.edu/

^2^ http://findlab.stanford.edu/research
